# The Shared Wildlife Health Information System (SWHIS): An Online Case Management System to Support Wildlife Management

**DOI:** 10.3390/ani16142180

**Published:** 2026-07-14

**Authors:** Sabrina S. Greening, Johannes Nelson, Julie C. Ellis

**Affiliations:** Wildlife Futures Program, Department of Pathobiology, New Bolton Center, University of Pennsylvania School of Veterinary Medicine, Kennett Square, PA 19348, USA

**Keywords:** wildlife health, databases, data standardization, disease surveillance, wildlife management

## Abstract

As emerging infectious diseases and human–wildlife interactions increasingly transcend political boundaries, the ability to detect patterns, compare data, and respond in a coordinated manner depends on the availability of interoperable, well-structured data systems. Wildlife health data are often collected by diverse stakeholders—including field biologists, wildlife rehabilitators, hunters, veterinarians, and diagnostic laboratories—using inconsistent methods, terminology, and levels of detail. Data stored in isolated systems results in duplication, information loss, and barriers to integration. This paper describes the design and development of the Shared Wildlife Health Information System (SWHIS), an online database system designed to facilitate wildlife health data management. Development incorporated continuous stakeholder feedback, including input from state wildlife management agencies, to ensure the system aligned with real-world workflows and user needs. Launched in October 2022, SWHIS is supported by a dedicated development team with a growing suite of tools to meet diverse user needs. Priority features under development include a mobile field application, a public reporting tool, and an event alerting system. By improving data standardization, accessibility, and long-term preservation, systems like the SWHIS strengthen wildlife health surveillance capacity and support timelier, evidence-based decision-making across jurisdictional boundaries.

## Simple Summary

The rise in emerging infectious diseases has made coordinated wildlife health surveillance more critical than ever. Wildlife health data are often collected by diverse stakeholders—including field biologists, wildlife rehabilitators, hunters, veterinarians, and diagnostic laboratories—but they use different methods and terminology, making it difficult to combine or compare data. The Shared Wildlife Health Information System (SWHIS) was developed to address this problem. SWHIS is an online database system that standardizes how wildlife health data are recorded and stored, making information easier to share, access, and preserve over time. The system was built with ongoing input from a range of users, including state wildlife management agencies, to ensure it works within real-world workflows. Launched in October 2022, SWHIS continues to grow. Features currently in development include a mobile app for fieldwork and an alert system for disease events. By bringing data together in a consistent, accessible format, systems like the SWHIS help wildlife health professionals manage and use their data in real time to help make better-informed decisions.

## 1. Introduction

An understanding of wildlife health and disease plays a crucial role in the identification and management of potential threats to both animal and human health [1,2]. Surveillance of wild animals is essential to understand the risks and impacts of diseases, pathogens, and toxins, as part of a One Health approach [3]. Traditional wildlife surveillance has largely been in reaction to adverse health events, such as mass mortalities, or relies on the opportunistic collection and sampling of sick or dead animals [4]. This type of surveillance draws on a wide range of data, including field observations, necropsy findings, diagnostic test results, and environmental information. Furthermore, data are often collected by multiple organizations or individuals, such as field biologists, wildlife rehabilitators, hunters, veterinarians, or diagnostic laboratories, using different methods, terminology, and levels of detail [5]. The diversity of data sources makes it difficult to ensure consistency and completeness across datasets. In many cases, wildlife management agencies rely on isolated systems, simple spreadsheets, or paper-based methods to compile health data without a unified structure, leading to duplication, information loss, and integration barriers [6,7]. For instance, data within a management agency are often siloed across species biologists who may use different terminology. When in reality, they often share the same habitats and therefore health threats.

The lack of centralized digital disease surveillance systems designed for wildlife health surveillance makes it difficult to address incongruency, provide timely access, ensure data sustainability. It also limits the ability to conduct analytics such as monitoring the health of populations, identifying emerging threats, tracking disease outbreaks, determining trends over time, or implementing targeted interventions [8,9,10]. Limited technical infrastructure and inconsistent data management practices across agencies also hinder timely data sharing and interoperability, especially when confidentiality or data ownership concerns arise. Additionally, resource constraints, such as inadequate funding, limited staffing, and a lack of data management expertise, can prevent regular updates, quality control, and maintenance of digital systems, lowering the capacity to conduct wildlife health surveillance [11].

Ongoing challenges to data management in wildlife health underscore the need for a centralized database system to support effective surveillance, research, and response to emerging health threats at the interface of wildlife, livestock, and humans [12]. A recent initiative by Schwantes et al. 2025, called for the inclusion of 40 data fields (9 required) and 24 metadata fields (7 required) with the goal of developing a standardized framework for recording, formatting, and sharing wildlife disease data [13]. However, this framework does not account for constraints often present in wildlife management agencies, including a lack of data management expertise and/or necessary resources to implement this approach.

One solution is to create a ready-to-use data management system that would provide agencies with a centralized, accessible, and secure platform where information from diverse sources can be compiled, standardized where feasible, and analyzed. Such a system would improve data quality and consistency by enforcing standardized entry fields, species taxonomies, and reporting formats, ensuring that information collected by different partners can be meaningfully compared and aggregated. It would also enable timely data sharing and collaboration among wildlife agencies, laboratories, researchers, and public health partners, facilitating timely analysis, fostering early detection and spatio-temporal clusters of unusual events, and supporting coordinated responses to disease outbreaks. Beyond immediate surveillance needs, an online database system allowing for the structured collection of digital information would ensure the long-term preservation of wildlife health records, safeguarding historical data that are often lost when stored in spreadsheets or individual institutional archives. By maintaining these records in a secure and structured system, agencies could track changes in disease occurrence over time, assess environmental or climatic influences, and evaluate the impact of management actions. Finally, an online system would enhance efficiency and transparency, automating reporting and visualization tools to support decision-making, communication with stakeholders, and the development of evidence-based policy. In this way, a well-designed wildlife health database system is not only a tool for data management but a critical foundation for protecting biodiversity, ecosystem health, and public well-being.

A number of wildlife health database systems already exist, such as the World Animal Health Information System (WAHIS; [14]), the Wildlife Health Intelligence Platform (WHIP; Canadian Wildlife Health Cooperative/University of Saskatchewan, Canada), the European Network of Wildlife Professionals (ENETWILD; enetwild.com), the Health and Wildlife Knowledge database (HAWK; [15]), and the Pathogen Harmonized Observatory (PHAROS; pharos.viralemergence.org), though they differ in both geographical scope and purpose. For instance, many of the databases that operate at the international or national level serve primarily as tools for disease reporting and data sharing across jurisdictions, and while these platforms provide valuable infrastructure for aggregating and reporting wildlife health information, they are generally designed for high-level situational awareness rather than the day-to-day operational needs of wildlife management agencies. Alternatively, some organizations have developed custom, in-house database systems tailored to their specific needs; however, these solutions are often limited by sustainability concerns, particularly the loss of institutional knowledge and technical capacity when key staff depart, as well as poor interoperability with other systems. SWHIS was designed to bridge this gap: unlike high-level international reporting platforms, it supports the granular, day-to-day data entry and case management needs of individual agencies, while unlike custom in-house systems, it is centrally maintained and supported by a dedicated development team, incorporates a standardized yet flexible data model, and is explicitly designed for interoperability with national systems.

This paper outlines the approach used to design and develop the Shared Wildlife Health Information System (SWHIS), an online database system that facilitates the management of wildlife health data. The system was launched in October 2022 and provides a user-friendly platform to record and visualize historic and ongoing wildlife health events. The system also provides mechanisms to manage wildlife diagnostic laboratory submissions and results, thereby enabling agencies to optimize staff time and resources by streamlining reporting. This paper aims to describe the structure of the SWHIS and how it could be used to support the management of wildlife health data, in addition to discussing the challenges faced during development and implementation, and future development plans and opportunities.

## 2. Materials and Methods

The development of the SWHIS began in October 2021, following a discovery period—a structured phase used to document user needs regarding wildlife health surveillance data management, existing workflows, data requirements, and system constraints—which could then be used to create a preliminary roadmap with prioritized features. Discovery was driven by the Wildlife Futures Program in collaboration with the Timmons Group Geospatial Solutions Division. The Wildlife Futures Program is a partnership between the University of Pennsylvania School of Veterinary Medicine and the Pennsylvania Game Commission. With expertise including wildlife veterinarians, ecologists, epidemiologists, data analysts, toxicologists, and pathologists/diagnosticians, the Wildlife Futures Program conducts wildlife health surveillance, research, and outreach to inform and improve wildlife management in Pennsylvania and beyond [16]. The Timmons Group provides advanced GIS services focusing on custom application development and strategic planning for smarter data-driven decisions [17].

A crucial first step during this period was to identify and engage with stakeholders, including state wildlife management agencies from across the U.S., the Association of Fish & Wildlife Agencies (AFWA) and its regional associations, U.S. Fish and Wildlife Service (USFWS), and the USGS National Wildlife Health Center (NWHC), to build a shared understanding of agency data challenges, needs, and priorities. We solicited input and feedback from these organizations through a variety of activities, including online interviews and surveys, which were used to develop (1) clear, predefined requirements that needed to be met for the database system to be considered complete and acceptable by stakeholders (i.e., acceptance criteria), (2) visualizations of different journeys/pathways a user may take when using the database system from beginning to end (i.e., user story maps), and (3) initial blueprints for the database system that outlined the structure/relational schema, content layout, and functionality (i.e., wireframes).

**Figure 1:**
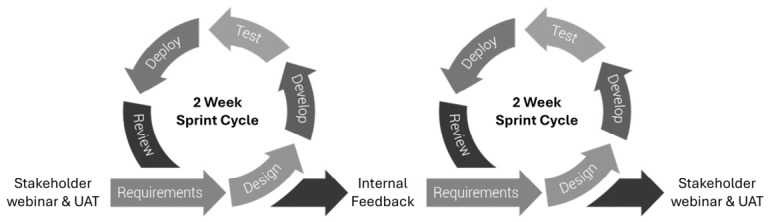
The modified agile development framework used during SWHIS development with two-week sprint cycles, monthly webinars and User Acceptance Testing (UAT), allowing for the integration of stakeholder feedback to refine features with minimal impact to the development timeline.

Throughout development, the SWHIS project followed a modified agile development framework (Figure 1), which emphasizes prioritization of high-value features for quicker delivery to users; transparent workflows with continuous demonstrations, feedback loops, and refinement; ongoing validation and stakeholder involvement to maintain momentum; and continuous refinement to ensure high-quality outcomes and adaptability to evolving needs. Similar agile development methods are widely used in the database and IT fields, as they offer a flexible, collaborative approach to software development that emphasizes iterative progress, rapid delivery, customer feedback, and continuous improvement [18,19]. This approach was supported by two-week “sprint cycles” that allowed for incremental changes to be made and tested frequently, rather than a single large release. In a traditional scrum practice, stakeholders would participate directly in sprint reviews at the end of each two-week cycle; however, in this modified approach, only members of the WFP reviewed changes every two weeks. For other stakeholders, monthly webinars and User Acceptance Testing (UAT) were conducted to gather real-time feedback that could be incorporated into the next sprint cycle. This modified structure reduced the workload for stakeholders and helped maintain high engagement as the development period continued over several months. The webinar sessions were also used as an opportunity to collaboratively develop a dynamic, prioritized list of needs, including features, fixes, and technical tasks (i.e., product backlog) that could be used after the system went live to prioritize site navigation, data model features, and new developments, in addition to breaking down the project scope into smaller chunks and setting transparent delivery milestones (i.e., release plan).

## 3. Results

### 3.1. SWHIS Data Model

The SWHIS uses a hierarchical structure with five levels: Event, Animal, Specimen, Test, and Results and Diagnoses. In this structure, each level is nested within the one above it (i.e., parent–child relationship), meaning that a single Event can contain multiple Animals, each Animal can have multiple Specimens, and so on. This structure was designed with input from state wildlife agencies to reflect the steps taken during the collection of wildlife health data, starting with a field investigation to sample collection, lab submission, and diagnostics. The Supplementary Materials shows an entity relationship diagram (ERD) that illustrates how each level relates to the other within the SWHIS and the data fields currently contained in each level. The workflow starts by creating an Event, which can be used to capture one or more animals that are epidemiologically linked. An Event can be classified into different event types to indicate the origins or purpose of the data (i.e., a mortality or morbidity event, capture-recapture, active, wildlife rehabilitation, public observation, research, or biobanking). Documents such as diagnostic PDF reports or photos that pertain to all animals within an event can be added at the Event and Animal level. The Animal level is nested under the Event level using a parent-to-child relationship. One or multiple animals can be recorded within an Event, with standardized data fields used to capture demographic information and clinical history, free text boxes for additional notes, and a repository for documents or photos. Similarly, the Specimen level is nested under the Animal level using a parent-to-child relationship, and one or multiple specimens can be added to each animal within an event. If a pooled sample has been taken, this can be indicated at the Specimen level with space to list any identifying information related to each sample that was used to make up the pool.

Nested under Specimens, one or multiple diagnostic tests can be selected. The list of available tests is controlled by a standardized table that was initially curated during the development process by combining tests from across four veterinary diagnostic labs in the U.S. In this model, necropsies are treated as a diagnostic test, meaning that gross pathology findings can be recorded using the same standardized result fields available for other tests. However, recognizing that necropsy data often require a greater level of detail and structure, a dedicated necropsy module is currently under development to allow findings to be captured in a more standardized and comprehensive way. The specific structure of this module, including whether it will incorporate a standardized injury coding system (e.g., lesion topography and morphology) or draw on existing anatomo-pathological glossaries, has not yet been finalized and will be determined as development progresses. New tests are validated by a member of the Wildlife Futures Program, and if the requested test already exists under a different name, the request will be added as a synonym to the existing test to maintain standard data entry. For each new test added, results can be recorded as confirmed positive, confirmed negative, detected/non-negative, not detected, inconclusive, not applicable, or other, with a free-text box to capture additional findings or methodological details for specific diagnostic tests (e.g., specific commercial kit, primers, or sensitivity and specificity of the assay used) and a repository for documents or photos.

For a diagnosis to be added to a record, it is not necessary to add a specimen or a test (i.e., a user can report a diagnosis that has not been confirmed by a test). A diagnosis can be added at the Event level, at the Animal level, or both. The list of available diagnoses is maintained similarly to the list of available tests; however, each diagnosis is also tagged with a parent diagnostic category. For example, if a diagnosis of pneumonia was provided, multiple parent categories are available such as “bacterial”, “viral”, or “fungal”. The user must select one parent category for the pneumonia diagnosis in order to save the record.

Across the SWHIS, adding new values to existing data fields, such as additional diagnostic tests, is controlled by the Wildlife Futures Program and can be done the same day once an email request has been made by a user. Adding new fields must be discussed between the user and the Wildlife Futures Program to understand the need and know how best to prioritize the addition in development. In addition to the controlled vocabulary for test names and diagnoses described above, the system incorporates standardized terminology across numerous other dimensions, including specimen type, container type, and specimen state. These vocabularies are actively maintained and expanded as new laboratories, agencies, and data sources are integrated into the system. Where possible, controlled vocabularies have been aligned with established external standards. Species names and taxonomic information are mapped to the Integrated Taxonomic Information System (ITIS), ensuring consistency with a widely used authoritative reference. Other administrative tables, such as the diagnostic test list, were built by consolidating terminology from across multiple existing sources; for example, the initial test list was compiled by merging test catalogues from four U.S. veterinary diagnostic laboratories. As new partners are onboarded and bring novel workflows or terminology, the corresponding controlled vocabularies are updated to ensure consistent data capture, semantic alignment, and interoperability across the platform. Where partner terminology does not directly match existing SWHIS vocabulary, terms can be added as synonyms to the relevant controlled vocabulary entries, allowing them to be correctly mapped during any extract-transform-load (ETL) processes. While this approach supports practical interoperability across current partners, full alignment with formal ontologies, such as the Veterinary Extension of SNOMED CT (VetSCT), is recognized as a longer-term goal that would further enhance semantic interoperability across systems.

**Figure 2:**
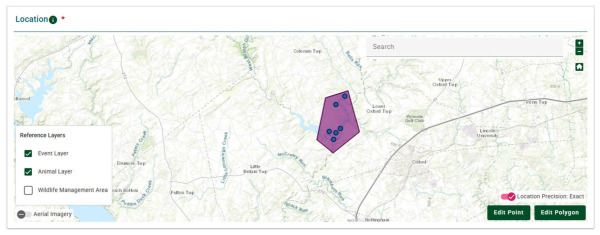
The SWHIS mapping interface displays the boundaries of a wildlife health event, indicated by the purple polygon (i.e., the event layer), and the locations of six animals, marked by blue circles (i.e., the animal layer). The red asterisk (*) indicates that the event location is a required field in the SWHIS entry form and must be completed before the record can be saved.

Geospatial data can be recorded at both the Event and Animal level. Users can identify a geographic location on the map interface (Figure 2) through two primary input methods: a text-based search (via a search bar) or direct interaction with the map by dropping a point or drawing a polygon. Both methods result in the system recording a GPS coordinate indicating the exact point location as provided by the user or a centroid position for any spatial unit, whether it is a defined polygon like an administrative boundary (e.g., county or township) or a geographic feature (e.g., a lake or a park). The user may also add their own geographical boundaries to the system, such as state-specific wildlife management areas, which are displayed on the map interface and will be recorded if the boundary intersects with the given location.

To maintain data quality throughout the system, SWHIS incorporates automated validation rules during both individual form entry and bulk data import, ensuring that only valid, correctly formatted data values are accepted into the system. Additionally, during bulk data import, SWHIS performs duplicate detection based on animal IDs to prevent redundant records from being introduced into the database. Together with the controlled vocabulary and standardized entry fields described above, these mechanisms support the ongoing accuracy and consistency of data captured within the SWHIS.

### 3.2. Hosting and Maintenance

Data are fully hosted using Amazon Web Services (AWS) cloud architecture. All data are kept securely in the cloud: attribute and location data are stored in an encrypted database (AWS Relational Data Store (RDS)), and photos and documents are stored in encrypted online storage (AWS S3 buckets), so everything is protected from unauthorized access. These data are served to the SWHIS application using Application Programming Interfaces (APIs) hosted on the cloud (AWS Lambda and API Gateway), allowing the app to safely receive the data when needed. Users entering and reading data must have a password-protected user account in AWS Cognito in order to access the API as well as the front-end web application using OAuth 2.0. User accounts in Cognito have an organization associated with them, which determines how data are filtered by the API so that each Organization only sees the data its users have entered into the SWHIS.

Organizations can choose to share their data with partner organizations through two mechanisms. Users can export records as an Excel spreadsheet directly from the SWHIS interface for use in external platforms or by sharing data directly within the SWHIS platform, which organizations manage themselves without requiring involvement from the Wildlife Futures Program. SWHIS provides the technical tools that enable this sharing but does not itself impose a formal permission hierarchy. Rather, each organization retains full control over whether, with whom, and to what extent its data are shared, consistent with the ownership principles established in the DUA. Decisions regarding time-limited or project-specific access are therefore managed directly between the sharing organizations rather than governed by SWHIS. When data are shared directly within SWHIS, the receiving organization can query and view the shared data but cannot edit or modify records, ensuring that data integrity and ownership remain with the originating organization. In addition to sharing with other organizations, an ArcGIS (ArcGIS Pro, Version 3.7, Redlands, CA, ESRI) feature service is available upon request, enabling organizations to directly import their SWHIS data into ArcGIS for spatial analysis and mapping.

### 3.3. Onboarding a New Organization or Agency

The onboarding process for the SWHIS is designed to ensure secure, consistent, and efficient integration of users and any historical data. SWHIS is a standard web-based platform accessible through any modern browser, requiring no special ports, VPN access, or software installation, which minimizes technical barriers for institutions with strict internal IT policies. There have been cases where automated system emails such as the temporary passwords sent during account creation have been blocked by an agency’s internal email security systems. For this reason, we advise organizations during onboarding to alert their IT department in advance to whitelist the sending address, which has effectively resolved this issue in practice. While SWHIS was developed primarily for use by U.S. wildlife management agencies, it is open to organizations internationally, though the platform is currently only available in English. Prospective organizations can request access to the SWHIS sandbox environment before onboarding as a way of testing the system without any further obligations or affecting the live production environment. If the organization decides to proceed, they are required to review and sign a Data Use Agreement (DUA) that outlines responsibilities regarding data governance, confidentiality, access, and permitted uses. Most critically, the DUA affirms that each organization retains full ownership of the data it contributes to the SWHIS; the Wildlife Futures Program does not claim ownership over agency data. The SWHIS DUA was developed in direct response to stakeholder concerns around data ownership, access, and misuse, and has been a crucial step in building trust with state agencies managing often sensitive data. Once the DUA has been completed, an organization account is created such that new users can select the organization they are associated with when signing up; however, before a new user can access the SWHIS, a designated administrator from the organization must approve the user and assign them to one of three roles: a read-only role that has permission to only view records, a read and write role that has permission to create, modify and view records, or an organizational administrator role with the same permissions as read and write users in addition to the ability to authenticate new users. A fourth role, the read-only limited role, provides further restricted access within an organization: users assigned this role have personal identifying information, location data, and any data related to forensic or police investigations hidden from their view. This ensures that sensitive records can be stored securely in SWHIS while still allowing only appropriately authorized staff to access them. Once enrolled, users will only have access to data entered in the SWHIS by themselves or other members within their own organization. As part of the onboarding process, organizations may choose to add historic data into the SWHIS with support available from Wildlife Futures staff to assist with data transformation, quality control checks, and secure transfer of historic datasets into the SWHIS. If an agency’s historical data are in the same format as the its current, ongoing data streams, it is possible that the transformation process can be developed into a continuous pipeline that automatically extracts from the agencies database, transforms the data into the SWHIS data model, and loads it into the SWHIS—reducing the need for double data entry on the part of the agency.

### 3.4. Ongoing Enhancements

New data fields can be added to the SWHIS based on user priorities, although we expect the data model to stabilize over time, such that the data fields remain relatively constant, and fewer updates are needed to the data tables that control SWHIS fields and terminology. To preserve the integrity of historic records, data fields are never removed from the system; if new fields are added, they will simply appear empty in any pre-existing records. For controlled vocabulary fields, if a primary value is updated or renamed, the previous value is retained as a synonym, ensuring it remains searchable and usable in filters. The updated value will be displayed to users going forward, while the synonym mapping ensures continuity for historic records and any ETL processes that rely on older terminology. Beyond the foundational data structure, there are several developments currently underway that aim to address different challenges previously identified by users. The first set of developments are part of a collaborative grant proposal led by the Caesar Kleberg Wildlife Research Institute (CKWRI) at Texas A&M University-Kingsville in partnership with the Texas Parks and Wildlife Department (TPWD). The project, entitled “National CWD Epidemiological Assessment” has several goals including developing a module in the SWHIS to help capture data specific to Chronic Wasting Disease (CWD) surveillance, building an ETL pipeline that utilizes an API to import diagnostic data in to the SWHIS directly from the Laboratory Information Management System (LIMS) used by Texas A&M Veterinary Medical Diagnostic Laboratory (TVMDL), building a second pipeline that would export data from the SWHIS to the CWD Data Warehouse—an online platform for CWD surveillance planning and data management (https://cwhl.vet.cornell.edu/project/sop4cwd, accessed on 7 July 2026), and developing epidemiological models within the Surveillance Optimization Project for Chronic Wasting Disease (SOP4CWD) to assess CWD spatial dynamics.

Another ongoing development includes the expansion of the SWHIS visualization dashboard. The new dashboard aims to provide enhanced data exploration and interactivity. Users will be able to switch between various map types, including choropleth maps and heat maps, to help identify spatial patterns. Search and filter functionality across all maps and visualizations will also allow users to refine graphics to help address certain research questions or fulfill specific reporting needs. Ideally, the dashboard will serve as the foundation for further action-oriented developments such as wildlife health alerts and other such data-driven insights. Lastly, the Wildlife Futures Program is collaborating with the PNNL on releasing a standardized lexicon for wildlife health databases, aiming to establish a common set of terms between the SWHIS and the National Wildlife Disease Database (NWDD) [20] to facilitate future integration between the two systems, as well as to promote standardization more widely in the field of wildlife health.

## 4. Discussion

The SWHIS has been developed as a data management tool to help wildlife management agencies with the day-to-day collection and management of wildlife health data. As of June 2026, 11 state agencies signed the DUA and members have collectively logged over 200,000 records in the SWHIS, including approximately 5700 surveillance events, 36,600 animals, 45,900 specimens, and 58,000 diagnostic results. A long-term goal is to help agencies make data-based disease management decisions as a crucial step towards the early detection and management of health threats for not only wildlife but also public health and livestock systems [2,21,22]. For agencies operating with a limited technical infrastructure, the SWHIS serves as a foundational structure to store and manage data in a way that promotes accuracy, interoperability, and accessibility across an organization. At its core, the system establishes a centralized data architecture, bringing together data generated from diverse sources and ensuring that information is consistent and usable for years to come. This centralization eliminates data silos, reduces duplication, and ensures that all contributors within an agency are working from a common, up-to-date dataset. It also supports controlled data access, allowing different users to view or contribute information according to their roles and permissions. The system emphasizes data standardization, enabling integration of datasets from different projects and regions, allowing reliable comparisons and large-scale analyses. Standardization also supports integration with other surveillance and reporting systems, such as the NWDD, and facilitates the expansion of the SWHIS, which primarily relies on form entry, to a system-to-system approach using APIs for data exchange between systems. Built-in tools for visualization, querying, and analysis further enhance usability, enabling users to generate maps, summaries, and reports in real time. These tools demonstrate the strengths of using stakeholder feedback to develop a database system, as it ensures the database is designed around real-world workflows and user needs, resulting in features that are practical and intuitive rather than theoretical. With continued feedback and a dedicated development team, the number of available tools in the system will also be able to grow over time to meet the needs of different users. Several priority features have been listed by users, including a field mobile app with offline capabilities, a public reporting tool, and an event alerting system. However, large-scale developments, such as these, require additional funding, which is often a significant and systemic challenge that hinders research, surveillance, and management efforts across wildlife health.

The funding strategy for the SWHIS and similar database systems is a crucial component of their sustainability. For many web resources, once project funding has expired, it can become difficult to maintain them [23]. To date, users have been able to access the system with no costs to them due to support of foundational grants. Currently, the Wildlife Futures Program is pursuing additional grants in the hope of maintaining the system and offering it to agencies at no additional cost. If this funding model is unsuccessful, the cost of the system will be split evenly across all the organizations, meaning that the cost will significantly reduce as the system grows and more agencies are onboarded. One challenge with this model is the uncertainty over the long-term system sustainability and how the system will be maintained, funded, and supported into the future, which increases stakeholder hesitancy. Stakeholder hesitancy has been reported across other data systems and often leads to the slow adoption of a new system [24,25].

One successful approach used to address stakeholder hesitancy in adopting the SWHIS was the establishment of a DUA that reassures agencies by clarifying responsibilities and ensuring all parties understand their obligations—building transparency and trust. In addition to concerns over sustainability, many potential users of the SWHIS also showed reluctance to participate fully due to concerns about data ownership, control, and the potential misuse or misinterpretation of their information. During development, many stakeholders expressed concern that once data are entered into a centralized system, they may lose authority over how those data are shared, credited, or analyzed. Others feared that sensitive information, such as the location of rare species or ongoing investigations, could be accessed or released inappropriately, leading to ecological, reputational, or regulatory risks. For these reasons, the DUA is of paramount importance to build trust and maintain transparency. For the SWHIS, organizations maintain ownership of their data and can only see data entered by users within their organization.

Another barrier to system adoption was the lack of a consistent data structure and organization within an agency. Depending on historical data management practices, existing datasets varied in their suitability for transformation into the SWHIS data model, and in many cases, agencies expressed concern over the condition of their data. In cases where data collection methods changed over time or standardized terminology was not used, substantial challenges arose. Addressing these issues often requires significant time and technical expertise—resources that are not always readily available to agencies, particularly for smaller agencies with few resources or competing priorities. This concern emphasizes the need for a more coordinated approach between wildlife surveillance programs, including the standardization of diagnostic protocols and the systematic collection of wildlife health data, as has been recognized previously [2,7,13]. Several agencies also expressed tension between adopting a general-purpose system and building a custom, agency-specific database, recognizing the appeal of a tailored solution while also acknowledging the significant upfront investment and long-term maintenance burden that such an approach requires. For many agencies, particularly smaller ones with limited resources, developing and sustaining a bespoke database system is simply not feasible. The SWHIS offers a practical alternative: a shared platform that can be continuously expanded to meet the specific needs of individual users, at a fraction of the cost of independent development. Critically, when a new feature is developed in response to the needs of one organization, it becomes available to all SWHIS users. This collaborative development model means that agencies collectively benefit from each other’s investments, and the system grows more capable over time in a way that no single agency could achieve alone.

To overcome barriers related to data transformation, the Wildlife Futures Program offers full technical support both for importing historic data and for ongoing data entry. The level of support provided is flexible and tailored to the needs and preferences of each organization. At one end of the spectrum, the Wildlife Futures Program can take a completely hands-off approach during the data cleaning process, with staff involvement limited to initiating the final data import once the organization has prepared its data. At the other end, the Wildlife Futures Program can manage the entire ETL process on behalf of the organization, from data extraction and transformation through to loading into the SWHIS. This support helps to bolster the technical capacity while reducing the workload requirements for users to get their data to match the SWHIS data model. Users can also provide feedback to the development team, highlighting future development opportunities to help ensure the system continues to meet their needs. The development team strives to maintain a balance between adapting the system to meet users’ specific needs and keeping the system generally useful across all agencies, only making alterations or additions if it is thought they will have a positive effect across multiple users. By streamlining data entry and offering ongoing technical support, the value of SWHIS can begin to be demonstrated to new users. Now that the SWHIS has passed the initial development phase, tangible outcomes, such as improved data quality and consistency, streamlined reporting, and real-time insights into wildlife health, reinforce the benefits of moving to a centralized database system. Regularly communicating these benefits, along with success stories or metrics showing impact, helps maintain motivation and fosters a sense of shared ownership among contributors, ultimately supporting the long-term sustainability of the database system.

A key goal of SWHIS is not to replace existing wildlife health data systems, but to function as part of a broader, interoperable ecosystem of platforms. It is widely recognized that no single system will ever capture all wildlife health data globally, nor should it. Different systems serve different purposes, user bases, and jurisdictions, and attempting to consolidate everything into one platform risks reducing usability and uptake. Instead, the priority should be ensuring that systems can seamlessly communicate and exchange data with one another—reducing duplication, enabling cross-platform analysis, and ultimately improving the flow of wildlife health information across institutional and jurisdictional boundaries. To this end, a data pipeline linking SWHIS with the SOP4CWD is currently under development, and a linkage with the USGS NWDD is a priority for future development. This collaboration serves as a valuable case study for the broader utility of the system: because SWHIS captures standardized demographic data (e.g., species, age, sex) and precise geospatial coordinates within its hierarchical data model, these key variables can be readily exported to support spatial epidemiological models of CWD dynamics developed through SOP4CWD. This capability illustrates how a well-structured, standardized data system can directly enable downstream analytical applications, and highlights the potential value of adopting similar systems for veterinary services in other states or countries seeking to support their own spatial disease modeling efforts. Beyond these specific integrations, additional linkages with other platforms can be built on request, reflecting the system’s commitment to interoperability as a core design principle rather than an afterthought. While there are currently no formal engagements planned with international organizations, the Wildlife Futures Program welcomes future data-sharing partnerships and is open to developing the necessary pipelines to support such collaborations. In this context, a comprehensive review of existing wildlife health data systems—comparing their scope, purpose, accessibility, and interoperability—would be a valuable resource for organizations navigating this landscape. Such a review would help agencies and researchers identify which platform, or combination of platforms, best suits their specific needs, and could highlight opportunities for greater coordination and integration across the field.

On a broader scale, the development of standardized wildlife health databases addresses a fundamental global challenge: the fragmentation of wildlife disease data across jurisdictions and institutions. As emerging infectious diseases and human–wildlife interactions increasingly transcend political boundaries, the ability to detect patterns, compare data, and respond in a coordinated manner depends on the availability of interoperable, well-structured data systems. Platforms such as the Shared Wildlife Health Information System provide a practical pathway toward this goal by enabling consistent data capture while respecting agency-level data ownership and operational constraints. By improving data standardization, accessibility, and long-term preservation, systems like SWHIS strengthen wildlife health surveillance capacity and support timelier, evidence-based decision-making.

## 5. Conclusions

By improving data standardization, accessibility, and long-term preservation, systems like the SWHIS strengthen wildlife health surveillance capacity and support timelier, evidence-based decision-making across jurisdictional boundaries. The SWHIS was developed in direct response to a well-documented gap in wildlife health data infrastructure: the lack of a centralized, standardized, and accessible system capable of meeting the day-to-day data management needs of wildlife agencies. Built with continuous stakeholder input, the system offers a flexible, disease- and species-agnostic platform that supports data entry, visualization, laboratory submission management, and cross-organizational data sharing, while ensuring that organizations retain full ownership and control of their data. Its hierarchical data model, controlled vocabularies, and role-based access controls provide a robust foundation for data quality and security, while its cloud-based architecture ensures long-term accessibility and scalability. Critically, SWHIS is designed not as a standalone solution but as part of a broader, interoperable ecosystem of wildlife health data platforms. By enabling seamless data exchange with other systems, fostering a collaborative development model in which new features benefit all users, and remaining open to partnerships at both national and international levels, SWHIS has the potential to meaningfully contribute to a more connected, resilient, and responsive global wildlife health surveillance network.

## Data Availability

The original contributions presented in this study are included in the article/Supplementary Materials. Further inquiries can be directed to the corresponding author.

## Supplementary Materials

The following supporting information can be downloaded at https://www.mdpi.com/article/10.3390/ani16142180/s1. Figure S1. An Entity-Relationship diagram (ERD) showing a visual representation of the hierarchical structure in the SWHIS with five levels/data entities: Event, Animal, Specimen, Test, and Results and Diagnoses. The key data fields are listed within each data entity, and the relationship between the different entities is represented with a solid black line, with one cardinal relationship, one-to-many, used between each step in the hierarchy (i.e., multiple animals can be recorded under one Event, and multiple specimens can be recorded under one *Animals*.

## Abbreviations

The following abbreviations are used in this manuscript:

SWHIS	Shared Wildlife Health Information System
WAHIS	World Animal Health Information System
WHIP	Wildlife Health Intelligence Platform
ENETWILD	European Network of Wildlife Professionals
HAWK	Health and Wildlife Knowledge database
PHAROS	Pathogen Harmonized Observatory
GIS	Geographic Information System
AFWA	Association of Fish & Wildlife Agencies
USFWS	U.S. Fish and Wildlife Service
NWHC	USGS National Wildlife Health Center
UAT	User Acceptance Testing
ERD	Entity-Relationship Diagram
ITIS	Integrated Taxonomic Information System
GPS	Global Positioning System
AWS	Amazon Web Services
RDS	Relational Data Store
API	Application Programming Interfaces
DUA	Data Use Agreement
CKWRI	Caesar Kleberg Wildlife Research Institute
TPWD	Texas Parks and Wildlife Department
CWD	Chronic Wasting Disease
ETL	Extract-Transform-Load
LIMS	Laboratory Information Management System
TVMDL	Texas A&M Veterinary Medical Diagnostic Laboratory
SOP4CWD	Surveillance Optimization Project for Chronic Wasting Disease
PNNL	Pacific Northwest National Laboratory
