# Supplementary material for: The Shared Wildlife Health Information System (SWHIS): An Online Case Management System to Support Wildlife Management
Source: Animals (Basel). 2026 Jul 14;16(14):2180. doi: 10.3390/ani16142180 (PMC13405183; doi:10.3390/ani16142180)
Supplement: Supplementary file 1 [file animals-16-02180-s001.zip › animals-4403161-supplementary.pdf]

# The Shared Wildlife Health Information System (SWHIS): An Online Case Management System to Support Wildlife Management

**Sabrina S. Greening * Johannes Nelson and Julie C. Ellis**

Wildlife Futures Program, Department of Pathobiology, Wildlife Futures Program, University of Pennsylvania School of Veterinary Medicine, Kennett Square, PA 19348, USA

* Correspondence: sgreenin@vet.upenn.edu

## Table of Contents

**Supplementary Figure S1.** An Entity-Relationship diagram (ERD) showing a visual representation of the hierarchical structure in the SWHIS with five levels/data entities: *Event*, *Animal*, *Specimen*, *Test*, and *Results* and *Diagnoses*. The key data fields are listed within each data entity, and the relationship between the different entities is represented with a solid black line, with one cardinal relationship, one-to-many, used between each step in the hierarchy (i.e., multiple animals can be recorded under one *Event*, and multiple specimens can be recorded under one *Animals*……………………………………………………………………………...**Pg. 2**

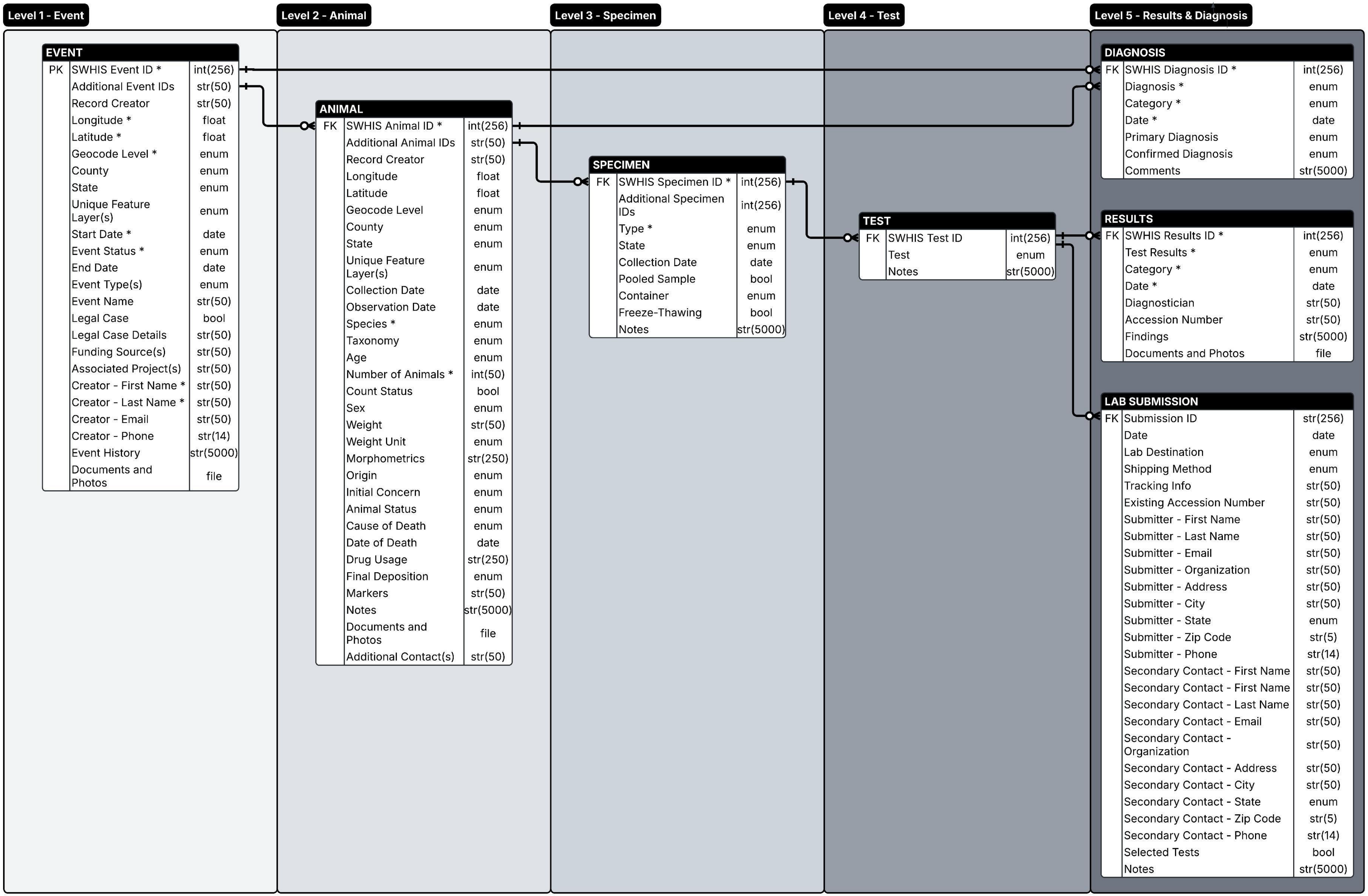
Level 1 - Event
Level 2 - Animal
Level 3 - Specimen
Level 4 - Test
Level 5 - Results & Diagnosis
EVENT
PK SWHIS Event ID * int(256)
Additional Event IDs str(50)
Record Creator str(50)
Longitude * float
Latitude * float
Geocode Level * enum
County enum
State enum
Unique Feature Layer(s) enum
Start Date * date
Event Status * enum
End Date date
Event Type(s) enum
Event Name str(50)
Legal Case bool
Legal Case Details str(50)
Funding Source(s) str(50)
Associated Project(s) str(50)
Creator - First Name * str(50)
Creator - Last Name * str(50)
Creator - Email str(50)
Creator - Phone str(14)
Event History str(5000)
Documents and Photos file
ANIMAL
FK SWHIS Animal ID * int(256)
Additional Animal IDs str(50)
Record Creator str(50)
Longitude float
Latitude float
Geocode Level enum
County enum
State enum
Unique Feature Layer(s) enum
Collection Date date
Observation Date date
Species * enum
Taxonomy enum
Age enum
Number of Animals * int(50)
Count Status bool
Sex enum
Weight str(50)
Weight Unit enum
Morphometrics str(250)
Origin enum
Initial Concern enum
Animal Status enum
Cause of Death enum
Date of Death date
Drug Usage str(250)
Final Deposition enum
Markers str(50)
Notes str(5000)
Documents and Photos file
Additional Contact(s) str(50)
SPECIMEN
FK SWHIS Specimen ID * int(256)
Additional Specimen IDs int(256)
Type * enum
State enum
Collection Date date
Pooled Sample bool
Container enum
Freeze-Thawing bool
Notes str(5000)
TEST
FK SWHIS Test ID int(256)
Test enum
Notes str(5000)
DIAGNOSIS
FK SWHIS Diagnosis ID * int(256)
Diagnosis * enum
Category * enum
Date * date
Primary Diagnosis enum
Confirmed Diagnosis enum
Comments str(5000)
RESULTS
FK SWHIS Results ID * int(256)
Test Results * enum
Category * enum
Date * date
Diagnostician str(50)
Accession Number str(50)
Findings str(5000)
Documents and Photos file
LAB SUBMISSION
FK Submission ID str(256)
Date date
Lab Destination enum
Shipping Method enum
Tracking Info str(50)
Existing Accession Number str(50)
Submitter - First Name str(50)
Submitter - Last Name str(50)
Submitter - Email str(50)
Submitter - Organization str(50)
Submitter - Address str(50)
Submitter - City str(50)
Submitter - State enum
Submitter - Zip Code str(5)
Submitter - Phone str(14)
Secondary Contact - First Name str(50)
Secondary Contact - First Name str(50)
Secondary Contact - Last Name str(50)
Secondary Contact - Email str(50)
Secondary Contact - Organization str(50)
Secondary Contact - Address str(50)
Secondary Contact - City str(50)
Secondary Contact - State enum
Secondary Contact - Zip Code str(5)
Secondary Contact - Phone str(14)
Selected Tests bool
Notes str(5000)
